# Sufficient Dimension Reduction: An Information-Theoretic Viewpoint

**DOI:** 10.3390/e24020167

**Published:** 2022-01-22

**Authors:** Debashis Ghosh

**Affiliations:** Department of Biostatistics and Informatics, Colorado School of Public Health, Aurora, CO 80045, USA; debashis.ghosh@cuanschutz.edu

**Keywords:** central subspace, information bottleneck, single-index model

## Abstract

There has been a lot of interest in sufficient dimension reduction (SDR) methodologies, as well as nonlinear extensions in the statistics literature. The SDR methodology has previously been motivated by several considerations: (a) finding data-driven subspaces that capture the essential facets of regression relationships; (b) analyzing data in a ‘model-free’ manner. In this article, we develop an approach to interpreting SDR techniques using information theory. Such a framework leads to a more assumption-lean understanding of what SDR methods do and also allows for some connections to results in the information theory literature.

## 1. Introduction

In statistical modeling, a key challenge is to determine appropriate transformations of the data that can reduce its dimension while at the same time capturing the essential information in the regression relationship between a set of covariates and a response variable. To this end, there has been a field of statistics, termed sufficient dimension reduction (SDR), that has sought to develop a methodology with this goal in mind. Broadly speaking, sufficient dimension reduction represents a class of ‘model-free’ methodologies that seek to find directions in the data that can capture the essential information in the regression relationship previously mentioned. An excellent recent monograph on the topic can be found in [1].

Historically, the basis for sufficient dimension reduction methods was the observation by authors, such as Brillinger [2] and Li and Duan [3], that regression parameters estimated by ordinary least squares were consistent, up to a constant, for their population counterparts in a generalized single-index model. This result required an assumption on the covariates being elliptically symmetric, which has been reframed into the current sufficient dimension reduction literature as the so-called linearity assumption. More recent formulations for sufficient dimension reduction have postulated the existence of a central subspace; subsequently, the goal of sufficient dimension reduction methods is to estimate the basis vectors of the central subspace. There now exists a wide variety of techniques available for estimation in sufficient dimension reduction; we will provide a review of such methods in Section 2.1.

In this article, we propose a new interpretation for sufficient dimension reduction based on conditional independence assumptions. Using graphical models, we are able to connect sufficient dimension reduction methods to information bottleneck theory [4]. The information bottleneck methodology was pioneered by the late Naftali Tishby and seeks to develop a ‘short code for X that preserves the maximum information about Y’ [4]. Information bottleneck typically formulates an optimization problem that seeks to find a compressed representation that minimizes information loss while imposing a penalty related to the expected distortion of the compression. The compression is the ‘bottleneck’ in the term ‘information bottleneck’. The optimization is solved using calculus of variations and leads to a set of self-consistent equations for finding optimal codes that are related to proposals by Blahut [5] and Arimoto [6]. The information bottleneck approach has been applied to a variety of problems in machine learning, such as document clustering [7,8], multivariate density estimation [9] and deep learning [10,11].

The interpretation developed in this paper allows us to demonstrate the following:

(a). We can view sufficient dimension reduction as a means of preserving information that is relevant to a response variable. It can be interpreted as performing the information bottleneck in two directions.
(b). Conversely, we will see that the information bottleneck is performing sufficient dimension reduction in a certain sense.
(c). By moving to mutual information, we can relax some of the distributional assumptions needed for sufficient dimension reduction in a manner different from that in [12,13,14,15,16]. This direction is a departure from the viewpoint that SDR serves as a means to estimate a target parameter, typically the span of basis vectors of the central subspace.
(d). In the case of Gaussian variables, we can develop a method for identifying ‘phase transitions’ in the structural dimension of central subspaces by expanding the work of [17] to handle sufficient dimension reduction procedures.

While many of the information-theoretic results are well-known to the information theory community, their embedding and merging with the literature on sufficient dimension reduction will be novel to statisticians.

The closest statistical work in nature to ours is that of Wang et al. [18]. They leverage the Hellinger integral of order two [19], which is related to the Kullback–Leibler divergence, an important quantity in information theory. Wang et al. [18] define subspace-based information measures using the Hellinger integral and demonstrate that a central subspace preserves information on this scale. For its estimation, they use a nonparametric regression approach that bears a resemblance to the minimum average variance estimation approach of Xia et al. [12]. The idea of using the Kullback–Leibler divergence for the optimization and estimation of the central subspace and other measures of association was used by Yin and co-authors in a series of papers [20,21,22,23]. We will also note work by Cook and Ni [24], who use a minimum discrepancy approach for finding the central subspace, and the work of Yao et al. [25], who developed a sufficient reduction procedure using the Fisher information metric, which can also be shown to be connected to Kullback–Leibler divergence. Finally, a device we use in the paper is graphical models, and we note recent work by [26].

The outline of this paper is as follows. We review the literature on sufficient dimension reduction, as well as pointing out some limitations, in Section 2. Section 3 seeks to develop connections between sufficient dimension reduction and information theory using graphical models. We focus on the Gaussian information bottleneck [17] and its relationship to SDR in Section 4. We illustrate the methodology with application to a dataset in Section 5. Section 6 concludes with some discussion.

## 2. Background and Preliminaries

### 2.1. Data Structures and Review of Dimension Reduction Methods

Much of the material presented here is expounded upon in the monograph by Li [1]. Let the data be represented as $\left(Y_{i},Z_{i}\right)$, i=1,…,n, a random sample from the joint distribution (Y,Z), where *Y* denotes the response of interest and *Z* is a *p*-dimensional vector of covariates. Suppose we formulate the following regression model for *Y* given *Z*:

(1) $$E\left(Y|Z\right)=g\left(\beta_{1}^{\prime }Z,\beta_{2}^{\prime }Z,\ldots ,\beta_{k}^{\prime }Z,u\right),$$

where $\beta_{j}$(j=1,…,k) are *p*-dimensional vectors of unknown regression coefficients, *u* is an error term, and *g* is an unspecified monotonic link function. Because of the presence of the parametric components involving $\beta_{j}$, as well as the nonparametric specification of the link function, model (1) is semiparametric. Note that when k=1, model (1) reduces to a single-index model [27]. In addition, model (1) can accommodate non-homoskedasticity in the error term if the variance depends on $\beta_{j}^{\prime }Z$.

The starting point of dimension reduction methods is the conditional independence of *Y* and *Z* given E(Y∣Z). We define two random variables, *A* and *B*, to be conditionally independent given *C* if

P(A|B,C)=P(A|C).

We will use the notation A⫫B|C to represent conditional independence. An implication of model (1) being true is that there exists a p×k matrix **B**, where

(2) $$Y⫫Z|B^{\prime }Z.$$

Another way of stating (2) is that the projection $B^{\prime }Z$ provides a sufficient data reduction and contains the essential information about the relationship between *Z* and *Y*. More generally, we can define a projection operator $P_{B}$ to be the symmetric and idempotent operator onto the subspace spanned by the columns of B. Then, (2) can be re-expressed as

(3) $$Y⫫Z|P_{B}Z.$$

If (3) holds, then it also holds for any subspace of $\tilde{B}$ such that the span of B is the same as the span of $\tilde{C}$. Let S(B) be the subspace generated by the columns of B. Let $S_{Y|Z}$ denote the intersection of all possible subspaces; if $S_{Y|Z}$ also satisfies (3), then we will refer to $S_{Y|Z}$ as the central subspace [28]. We will assume throughout that the central subspace exists [28,29,30]. In the classical presentation for sufficient dimension reduction methodology, the parameter has been defined to be the span of $S_{Y|Z}$. In other words, if $v_{1},\ldots ,v_{K}$ denote the basis vectors for $S_{Y|Z}$, then

$$S_{Y|Z}\equiv \mathrm{span}\left(v_{1},\ldots ,v_{K}\right)$$

is the target of sufficient dimension reduction procedures. Thus, there is an estimand that is often targeted by sufficient dimension reduction procedures.

We assume, without a loss of generality, that *Z* has a mean zero vector and covariance matrix equal to the identity matrix. One key assumption necessary for the implementation of one class of sufficient dimension reduction procedures is that the distribution of *Z*, conditional on $P_{B}Z$, satisfies a conditional linearity in the mean, i.e.,

(4) $$E\left(Z|P_{B}Z\right)=P_{B}Z.$$

Assumption (4) pertains to the marginal distribution of *Z* and means that all the information about *Z* is contained in its projection onto the subspace spanned by B. One class of distributions that satisfies the linearity condition is the family of elliptically symmetric distributions. This includes distributions, such as the multivariate normal distributions and scale mixtures of multivariate normal distributions.

As mentioned in the Introduction, there are many algorithms available for estimating the basis vectors of the central subspace. We describe the implementation of sliced inverse regression proposed by Li [28].

(a). ‘Slice’ the response variable *Y* into *J* slices, denoted as $Y_{1},\ldots ,Y_{J}$;
(b). Standardize the predictor observations as
  $${\tilde{Z}}_{i}={\hat{\Sigma }}^{-1/2}\left(Z_{i}-\hat{\mu }\right),\left(i=1,\ldots ,n\right),$$
  where $\hat{\mu }$ and $\hat{\Sigma }$ are the sample mean and covariance matrices of $Z_{1},\ldots ,Z_{n}$;
(c). Calculate sample mean estimates within slices: ${\bar{Z}}_{j}={n_{j}}^{-1}\sum_{i=1}^{n}I\left(Y_{i}\in Y_{j}\right){\tilde{Z}}_{i},$ where $n_{j}=\sum_{i=1}^{n}I\left(Y_{i}\in Y_{j}\right)$, j=1,…,J;
(d). Estimate the population covariance matrix of *Z* given *Y* by
  $$\hat{\Theta }=\sum_{j=1}^{J}\frac{n_{j}}{n}{\bar{Z}}_{j}{\bar{Z}}_{j}^{\prime };$$
(e). Compute the eigenvalues of $\hat{\Theta }$. These are the estimates of the basis vectors for the central subspace.

This algorithm is termed ‘inverse regression’ because effectively, information on the ‘backwards regression’ E(Z∣Y) is being estimated here rather than the ‘forward regression’ E(Y∣Z). Li [28] argues that this approach circumvents the usual issue of the curse of dimensionality. Other advantages of the sliced inverse regression algorithm are that it avoids multivariate nonparametric smoothing and is quite easy to fit.

The validity of the sliced inverse regression algorithm for estimating the central subspace relies on the linearity assumption. There has been much work on developing alternative estimation procedures that seek to relax the linearity assumption. For example, Xia et al. [12] propose the minimum average variance estimation procedure, which relies on a combination of nonparametric smoothing with weighted least squares. Since it involves nonparametric regression, its convergence depends on an appropriate rate of convergence for the bandwidth in conjunction with the sample size converging to infinity. Cook and Ni [24] proposed a minimum discrepancy method in which sufficient dimension reduction is characterized using an objective function approach. This leads to an alternating least squares algorithm for the estimation of the central subspace.

Many of the sufficient dimension-reduction methods can be viewed as solving the following eigenvalue/eigenvector problem:

(5) $$Ab_{j}=\lambda_{j}\Sigma_{Z}b_{j},$$

for j=1,…,k, where $\Sigma_{Z}$ denotes the covariance matrix of *Z*, and $\left(\lambda_{j},b_{j}\right)$ denotes the eigenvalue/eigenvector pairs. We say that the matrix pair $\left(A,\Sigma_{Z}\right)$ is a generalized eigenvalue solution (GES) if it satisfies (5). This is discussed at length in the monograph by Li [1]. Note that typically, the solutions to (5) are returned as

$$\lambda_{1}\geq \lambda_{2}\geq \cdots \geq \lambda_{k},$$

and $b_{j}^{\prime }\Sigma_{Z}b_{j}=1$ for j=1,…,k. The choice of A in (5) depends on the particular sufficient dimension reduction algorithm that is used. For example, in sliced inverse regression, A would represent the covariance matrix of the slice means. For principal Hessians directions [31], A in (5) is taken to be a weighted covariance matrix of the response to *Z*.

The matrix formulation in (5) allows for immediate generalizations to nonlinear versions of sufficient dimension. This can be done by replacing **A** in (5) with a so-called ‘kernelized’ matrix computed using inner products of covariates mapped to higher-dimensional spaces. Such methods are related to the procedures in Wu et al. [32], Fukumizu et al. [14] and Lee et al. [16].

### 2.2. Limitations of Sufficient Dimension Reduction

As mentioned above, one of the key assumptions in applying sufficient dimension reduction methodology is termed the linearity condition. A sufficient condition for this to hold is that the predictor variables of interest follow an elliptically contoured distribution. Distributions that satisfy elliptical symmetry include the multivariate normal distribution and the multivariate *t*-distribution. One of the main criticisms leveled against the sufficient dimension reduction methods is that this assumption will not be satisfied in practice. For example, if covariates are discrete, then this will violate the linearity condition. Many authors invoke the theoretical results of Hall and Li [33], which suggests that in an asymptotic framework, the linearity condition will hold. An alternative approach has been to develop generalizations of the sufficient dimension reduction methodology that relax the linearity condition. Such approaches can be found in proposals, such as Chiaromonte et al. [34], Fukumizu et al. [13,14], Li et al. [15] and Lee et al. [16].

The other issue with sufficient dimension reduction methods involves the identification of the basis of $S_{Y|Z}$, which is referred to as the directions of the central subspace. These vectors are not estimable in the situation where the components of *Z* are discrete. Such variables arise routinely in biomedical, sociological and demographic studies (e.g., race/gender), and this limitation makes the use of sufficient dimension reduction methods challenging. In an important work, Chiaromonte et al. [34] developed an approach to sufficient dimension reduction with categorical predictors. The idea is to perform the sliced averaging of the continuous covariates within each of the levels defined by the combination of the categorical variables. Then, the level-specific covariance matrices are pooled, and the directions are estimated using spectral decomposition, similar to the description of sliced inverse regression in Section 2.1.

## 3. Graphical Models, Connections and Information Theoretic Results

To link sufficient dimension reduction methods to the information bottleneck, we will now introduce some concepts from graph theory and graphical models [26,35]. A graph G=(V,E) consists of a set of vertices *V* and a collection of edges *E*. Here, $V\equiv \{v_{1},\ldots ,v_{m}\}$ denotes the collection of *m* vertices and the edges *E* consist of two-element subsets of the power set of *V* that denote edges between vertices. To simplify the discussion, we will assume that there are no edges from a vertex to itself, i.e., no self-loops. Graphs whose edges have more than two elements are referred to as hypergraphs [36] and will not be considered further in the paper. The vertices represent random variables, and the edges are used to specify dependencies between the random variables. There are two types of edges we will consider here between vertices $v_{1}$ and $v_{2}$. A directed edge is denoted by $v_{1}\to v_{2}$ and implies that $v_{1}$ affects $v_{2}$ and not vice versa. An undirected edge is denoted by $v_{1}-v_{2}$ and is equivalent to $v_{1}\to v_{2}$ and $v_{2}\to v_{1}$. Thus for undirected edges, $v_{1}$ and $v_{2}$ simultaneously affect each other. We define the parents of a vertex *v* by

pa(v)={u∈V:∃adirectededgefromutov}.

It is a well-known fact that for an acyclic directed graph [26,35], one can factorize the joint distribution of random variables defined on the graph *G* as

(6) $$f\left(X_{u}:u\in G\right)=\prod_{v\in V}p\left(x_{v}|x_{s},\;s\in pa\left(v\right)\right).$$

The final graphical model concept we will need is that of d-separation [35]. If G is a directed graph in which *X*, *Y* and *Z*. are a disjoint sets of vertices, then *X* and *Y* are d-separated by *Z* in *G* if and only if every path from a vertex in *X* to a vertex in *Y* is intercepted by a vertex in *Z*.

We can see that assumption (3) corresponds to the following graphs

(7) $$\begin{array}{ccc} Z & \to P_{B}Z & \to Y\\ Z & \leftarrow P_{B}Z & \leftarrow Y\\ Z & \to P_{B}Z & \leftarrow Y\\ Z & \leftarrow P_{B}Z & \to Y \end{array}$$

This follows from using the definition of undirected graphs and conditional independence. Similarly, the information bottleneck approach works with the graph

(8) Z→T→Y

The comparison of (7) and (8) offers the following insights. First, the central subspace performs d-separation of *Z* and *Y*. Similarly, the role of *T* in the information bottleneck framework is to intercept paths between *Z* and *Y*. This leads us to the following result, which will be new to statisticians:

**Proposition** **1.** *The central subspace can also serve as an information bottleneck.*

**Proof.**  The proof of the proposition follows by observing that the graph in (8) is a subgraph of the graphs in (7). □

**Remark** **1.** *Returning to the work of Wang et al. [18], the graphical representation in (7) makes sufficient dimension reduction integrating the forward and backward regressions. The graphs in (7) are precisely the forward and backward regression that Wang and colleagues speak of. They can also be viewed as ‘forward’ and ‘backward’ information bottlenecks. Thus, we see that sufficient dimension reduction is attempting to simultaneously perform a forward and reverse information bottleneck, while information bottlenecks themselves operate in the forward direction.*

*Based on the proposition, we observe that the role of the central subspace in sufficient dimension reduction plays a role akin to the information bottleneck. Using the viewpoint of information theory, we can interpret the goal of sufficient dimension reduction as one of information compression. This allows the use of these methods even in situations when the central subspace will not be estimable.*

To make the idea concrete, we will be interpreting $P_{B}Z$ as a random variable in the rest of the section. We will further assume that *Z* and *Y* are discrete random variables that are potentially multivariate. The entropy of *Z* is defined by

(9) $$H\left(Z\right)=-\log\sum_{z\in Z}p\left(z\right)\log p\left(z\right),$$

where Z is the range of *Z* and p(z) denotes the probability mass function. Similarly, we can define the mutual information of *Z* and *Y* as

(10) $$I\left(Z;Y\right)=\sum_{z,y}p\left(z,y\right)\log\frac{p(z,y)}{p(z)p(y)}$$

which extends upon (9) in a natural way. Mutual information measures the dependence between two random variables. It has the following properties: (a) it is symmetric in *Z* and *Y*; (b) it is nonnegative; (c) it is equal to zero if and only if *Z* and *Y* are independent.

A comprehensive overview of entropy and mutual information can be found in Cover and Thomas [37]. To keep the discussion self-contained, we now provide a summary of many basic properties of entropy and mutual information. Further details can be found in Chapter 2 of Cover and Thomas [37].

**Property** **1.**

*1.*  *I(Z;Y)=H(Z)−H(Z|Y), where $H\left(Z|Y\right)=-\sum_{z,y}p\left(z,y\right)\log p\left(z|y\right)$.*
*2.*  *I(Z;Y)=H(Y)−H(Y|Z).*
*3.*  *I(Z;Y)=I(Y;Z).*
*4*  *I(Z;Y)=H(Z)+H(Y)−H(X,Y).*
*5.*  *H(X,Y)=H(X)+H(Y|X).*

We note that the last property is typically referred to as the chain rule for entropy and can be extended to more than two random variables.

For the graphs considered in (7) and (8), we need to consider conditional versions of mutual information. This is given to us by Equation (2.61) of Cover and Thomas [37].

**Definition** **1.** *The conditional mutual information of Z and Y given W is defined as*

$$I\left(Z;Y|W\right)=\sum_{z,y,w}p\left(z,y,w\right)\log\frac{p(z,y|w)}{p(z|w)p(y|w)}.$$

*Finally, we will need one more definition from Chapter 2.8 of Cover and Thomas [37].*

**Definition** **2** (p. 34 of [37]). *Z,Y and W form a Markov Chain, denoted as Z→W→Y if the conditional distribution of Y given W and Z only depends on Z.*

We assume a reversible Markov Chain so that Z→W→Y and Y→W→Z are treated as equivalent. Thus, the reversibility of the Markov Chain allows us to conceptually drop the directionality in DAGs, which becomes in line with the conditional independence assumptions outlined in Section 2.1. We have the following celebrated result from information theory, the data-processing inequality (p. 34 of [37]):

**Theorem** **1.** *If Z→W→Y, then I(W;Z)≥I(Y;Z).*

The data processing inequality guarantees equality if and only if *Z* and *Y* are independent given *W*. We can now take these results and apply them to the graphs for sufficient dimension reduction.

**Corollary** **1.** *Assumption (3) is equivalent to $I\left(Z;P_{B}Z\right)=I\left(Z;Y\right).$ This corollary also relates to Theorem 1 of Wang et al. [18]. This formalizes the proposition earlier in the paper.*

**Remark** **2.** *Note that we have rephrased the central subspace as a random variable that attempts to minimize an information-based criterion. Thus, we get away from the traditional viewpoint where we view the goal of sufficient dimension reduction as targeting the span of the central subspace. Doing so provides another justification for the use of sufficient dimension reduction. This is in the spirit of ‘assumption-lean inference’ [38] in which the goal is to have available statistical methods that can be useful even when a true model or parameter does not exist.*

**Remark** **3.** *The mutual information is intimately related to the Kullback–Leibler divergence of two probability measures. A nice overview of how Kullback–Leibler divergences are related to information theoretic quantities can be found in [19]. We will explore the link between sufficient dimension reduction methods and Kullback–Leibler divergences in future work.*

## 4. The Case of Gaussian Variables

In most problems involving the information bottleneck, one can use the Blahut–Arimoto algorithm [5,6], which is an iterative algorithm that involves repeated projection operations. In this section, we study a noniterative information bottleneck algorithm by Chechik et al. [17]. They deal with the situation of *Z* and *Y* having a joint Gaussian distribution and show that one can use an eigenvector/eigenvalue decomposition of certain matrices to achieve an information bottleneck. We then show how to relate this to several sufficient dimension reduction procedures.

Chechik et al. [17] considered the situation of (Z,Y) having a joint Gaussian or multivariate normal distribution. Without loss of generality, we will assume a mean of zero throughout the section. The goal of the Gaussian information bottleneck is to find a mapping from *Z* to *T*, such that the information content of *Z* is sufficiently compressed while at the same time maintaining its association with *Y*. Formally, the Gaussian information bottleneck involves the minimization of

L≡I(Z;T)−βI(T;Y)

over matrices A and $\Sigma_{e}$, where

(11) $$T=AZ+e,\;\;\;e∼MVN(0,\Sigma_{e}),$$

and MVN(0,Σ) denotes a multivariate normal distribution with mean zero vector and covariance matrix Σ. Note that in (11), we assume that *e* is independent of *Z*. Because of the linearity of *T* in *Z* in (11), *T* will have a multivariate Gaussian distribution with mean zero vector and covariance matrix $A\Sigma_{Z}A^{\prime }+\Sigma_{e}$. Chechik et al. [17] prove the following theorem.

**Theorem** **2** (Theorem 3.1. of [17]). *The optimal solution to the Gausssian information bottleneck problem (11) for a given β is given by $\Sigma_{e}^{opt}=I$ and*

$$A=\left\{\begin{array}{c} \left[0^{\prime }\cdots 0^{\prime }\right]\;if\;0\leq \beta \leq \beta_{1}^{*}\\ {}[\gamma_{1}v_{1}^{\prime }\;\;0^{\prime }\cdots ]\;if\;\beta_{1}^{*}<\beta \leq \beta_{2}^{*}\\ {}[\gamma_{1}v_{1}^{\prime }\;\;\gamma_{2}v_{2}^{\prime }\;\;0^{\prime }\cdots ]\;if\;\beta_{2}^{*}<\beta \leq \beta_{3}^{*}\\ \vdots \end{array}\right.$$

*where $\gamma_{1},\ldots ,\gamma_{n}$ are functions of the eigenvalues $\alpha_{1},\ldots ,\alpha_{n}$ of $\Sigma_{Z|Y}\Sigma_{Z}^{-1}$, defined as*

$$\gamma_{i}=\sqrt{\frac{\beta (1-\alpha_{i})-1}{\alpha_{i}r_{i}}},$$

*$r_{i}=v_{i}^{\prime }\Sigma_{Z}v_{i}$ and*

$$\beta_{i}^{*}={\left(1-\alpha_{i}\right)}^{-1},i=1,\ldots ,n.$$

Thus, the theorem demonstrates the tradeoff between compression and its associated cost. If β is smaller than $\beta_{1}^{*}$, then the cost is too high, and the optimal solution is the zero matrix. Otherwise, we see that we can start to identify subspaces for larger values of β associated with the eigenvectors of $\Sigma_{Z|Y}\Sigma_{Z}^{-1}$. We also see a transition in terms of the dimensions of the subspaces spanned by $v_{i}$ as β increases. There are also discrete jump points for β. We refer to the result of Theorem 2 as the Gaussian Information Bottleneck Theorem.

Note that the theorem also involves solving the eigenvalue/eigenvector decomposition via the equation

$$\Sigma_{Z|Y}v=\lambda \Sigma_{Z}v.$$

Comparing the structure of this equation to (5), we see that $\left(\Sigma_{Z|Y},\Sigma_{Z}\right)$ can be viewed as a GES. The difference between the typical GES solution with the result of Theorem 2 is the order in which eigenvalues appear. For GES, they occur in descending order, while for Theorem 2, they are in ascending order. Using the link of Theorem 2 to generalized eigenvector solutions, we have the following result for sufficient dimension reduction methodology [17] to demonstrate the following result.

**Proposition** **2.** *The result of the Gaussian information bottleneck theorem holds*

*(a).*  *for sliced inverse regression with $Cov\{E\left(Z|Y\right),\Sigma_{Z}\}$ as a GES;*
*(b).*  *for partial inverse regression with $Cov\{\Sigma_{ZF}\Sigma_{FF}^{-1}\Sigma_{FZ},\Sigma_{Z}\}$ as a GES, where for a known transformation of Y, F(Y),*
$$\begin{array}{ccc} \Sigma_{FF} & = & VarF(Y)\\ \Sigma_{FZ} & = & Cov\{F(Y),Z\} \end{array}$$
*(c).*  *for sliced average variance estimation [12] with $Cov\{\Sigma_{Z}-Var\left(Z|Y\right),\Sigma_{Z}\}$ as a GES;*
*(d).*  *for principal Hessians directions [31] with $Cov\{\Sigma_{ZZY},\Sigma_{Z}\}$ as a GES, where*
$$\Sigma_{ZZY}=E\left(ZZ^{\prime }Y\right)$$

**Proof.**  All of these results follow by defining the GES equivalences as found in Li [1]. □

The proposition affords us new insights into how to view the information compression/basis calculation for several existing sufficient dimension reduction procedures from the information bottleneck viewpoint.

We note that if we sort the eigenvectors in descending order, the problem of selecting which index to stop is precisely that of selecting the dimension of the central subspace. This is an important problem for which there have been several approaches in the literature. Ye and Weiss [39] proposed an approach to the selection using the nonparametric bootstrap. Recently, Luo and Li [40] proposed the ladle approach, which used the bootstrap but combined information from both the eigenvalues and the eigenvectors of the central subspace to determine the dimension of the central subspace. Another recent innovation by Luo and Li [41] was to augment the predictor matrix with noise variables, which is in the spirit of the recent, popular ‘knockoffs’ approach in statistics [42]. One sees that the problem of order determination of the central subspace is dual to the Gaussian information bottleneck theorem. Equivalently, increasing the dimension of the central subspace will be orthogonal to the goal of minimizing information compression.

## 5. Numerical Illustration

The example in this paper comes from a randomized trial of opioid-dependent participants. Opioid addiction involving both heroin and diverted prescription opioid use represents major public health epidemics in the United States [43]. Currently, two treatments that are effective for opioid addition are agonist therapy with either buprenorphine (BUP) or methadone (MET). The study by Saxon et al. [44] was to determine if there were differences between BUP and MET with respect to liver function in subjects being treated for opioid dependence. Subjects who met the study inclusion criteria were randomized to BUP or MET; there was a total of n=832 subjects in the analysis. Here, we will focus on the change in weight from baseline to week 12 as the dependent variable. Predictor variables include weight at baseline, treatment, gender and ethnicity. Assuming that the central subspace is of dimension one, using sliced inverse regression [28], we estimate the basis to be (−0.38,−0.75,0.53,0.03). Thus, we would estimate the first direction to be

(12) −0.38Tx−0.75Gender+0.53Ethn+0.03BaseWt.

Note that in the classical framework of sufficient dimension reduction, the interpretation of the estimate is problematic. This is due to the fact that treatment, gender and ethnicity are binary variables. This means that viewed as an estimand; the central subspace formally does not exist. Having said this, the framework in this paper would view (12) as the linear combination of the variables that achieves maximum information compression in the predictors while simultaneously minimizing information loss between the covariates with the outcome variable. Note that this interpretation does not require the existence of a central subspace.

Naik and Tsai [45] proposed the use of partial least squares (PLS) as a means of sufficient dimension reduction in the situation where the dimension of the central subspace equals one. For these data, we would estimate a combination analogous to (12) based on partial least squares by

(13) 0.0001Tx+0.0003Gender−0.0001Ethn−0.0268BaseWt

Comparing the magnitudes of (12) and (13), SIR estimates larger relative weights with the exception of weight at baseline. Again, we can interpret the PLS estimate as the linear combination of the variables that achieves maximum information compression in the covariates while simultaneously minimizing information loss regarding their association with the outcome variable. A Github repository illustrating these analyses can be found at http://github.com/GhoshLab/ITSDR/.

## 6. Discussion

In this article, we have attempted to reinterpret the sufficient dimension reduction methodology in the statistical literature using connections to information theory. This link, and in particular to that of the theory of information bottleneck [4], allows for some new insights and interpretations to occur:

1. We can avoid the goal of SDR as estimating a parameter, namely the basis of the central subspace, and view it instead as a means for information compression while simultaneously preserving association with an outcome variable. This information-theoretic view can allow for one to relax distributional assumptions in a way that is different from the σ—field approach described in [16].
2. By recognizing that the Gaussian bottleneck information theorem (Theorem 3.1 of [17]) is identical to solving a generalized eigenvalue problem, we can extend the results of [17] to a variety of sufficient dimension reduction methods. There, we see that the goals of information compression and central subspace dimension estimation are dual to each other.

Our hope is that this initial exploration of information theory with sufficient dimension reduction will allow for the adaptation and extension of information theoretic concepts into the SDR literature. We envision there being connections and development of methodologies for SDR in time series [46] and online [47,48] problems. This is currently under investigation.
